# (±)-Zanthonitidine A, a Pair of Enantiomeric Furoquinoline Alkaloids from *Zanthoxylum nitidum* with Antibacterial Activity

**DOI:** 10.1007/s13659-018-0169-7

**Published:** 2018-05-31

**Authors:** Li-Na Zhao, Xi-Xi Guo, Shuai Liu, Li Feng, Qi-Rui Bi, Zhe Wang, Ning-Hua Tan

**Affiliations:** 1 Department of TCMs Pharmaceuticals, School of Traditional Chinese Pharmacy, China Pharmaceutical University, Nanjing, 211198 China; 2 Faculty of Life Science and Food Engineering, Huaiyin Institute of Technology, Huaian, 223001 China

**Keywords:** *Zanthoxylum nitidum*, Furoquinoline alkaloids, Zanthonitidine A, Antibacterial activity

## Abstract

**Abstract:**

A pair of new enantiomeric furoquinoline alkaloids, (±)-zanthonitidine A (**1**), together with nine known ones (**2**–**10**) were isolated from the radix of *Zanthoxylum nitidum*. Their chemical structures were elucidated based on the extensive spectroscopic analysis. The racemic mixture of **1** was separated by chiral column chromatography, and the absolute configurations of (+)-**1** and (−)-**1** were determined by the comparison of experimental and calculated electronic circular dichroism spectra. Antibacterial activities of compounds **1**–**9** were evaluated, and compounds (+)-**1**, (−)-**1**, **3**, **7** and **8** showed antibacterial activities against *Bacillus subtilis*, *Enterococcus faecalis* or *Staphylococcus aureus*.

**Graphical Abstract:**

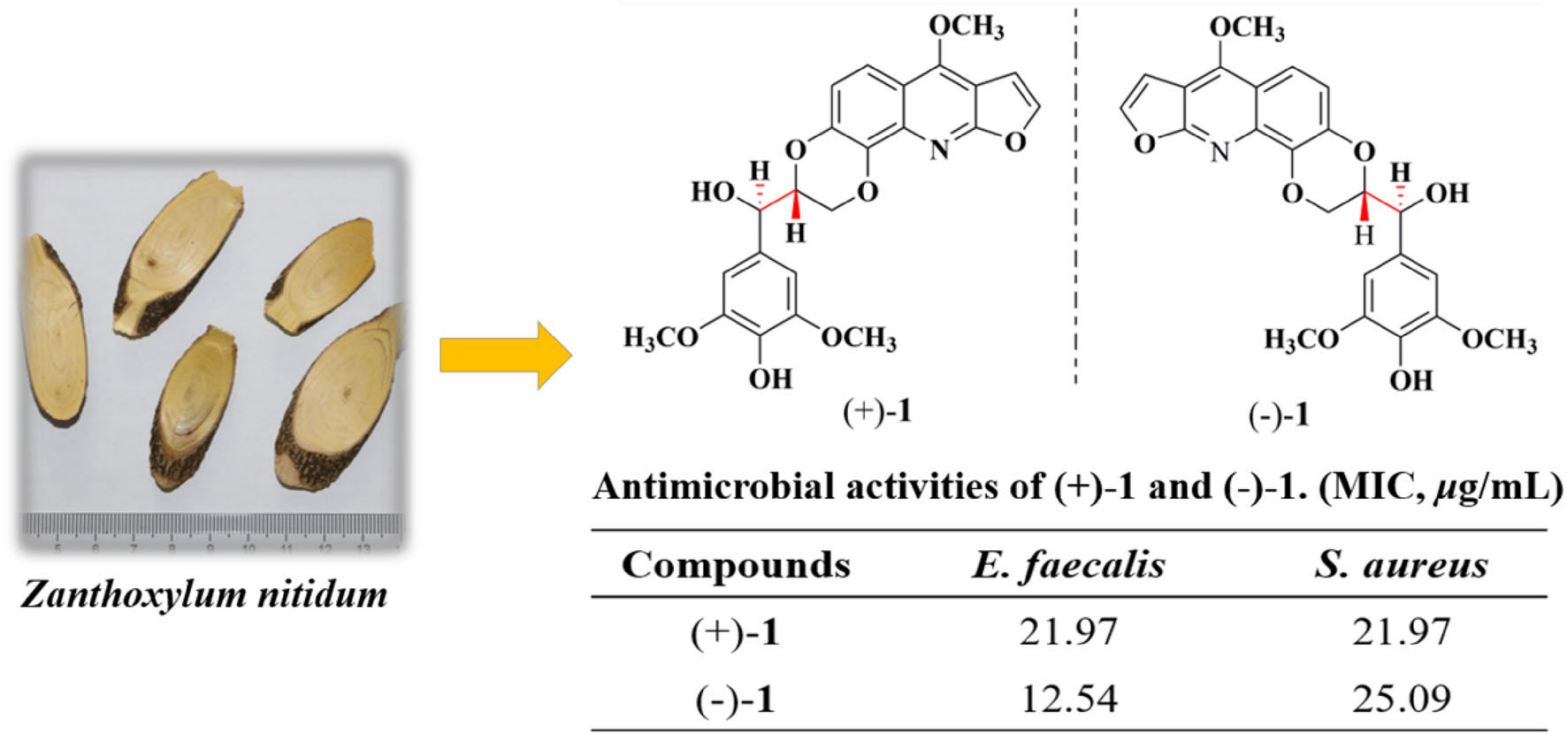

**Electronic supplementary material:**

The online version of this article (10.1007/s13659-018-0169-7) contains supplementary material, which is available to authorized users.

## Introduction

**Fig. 1:**
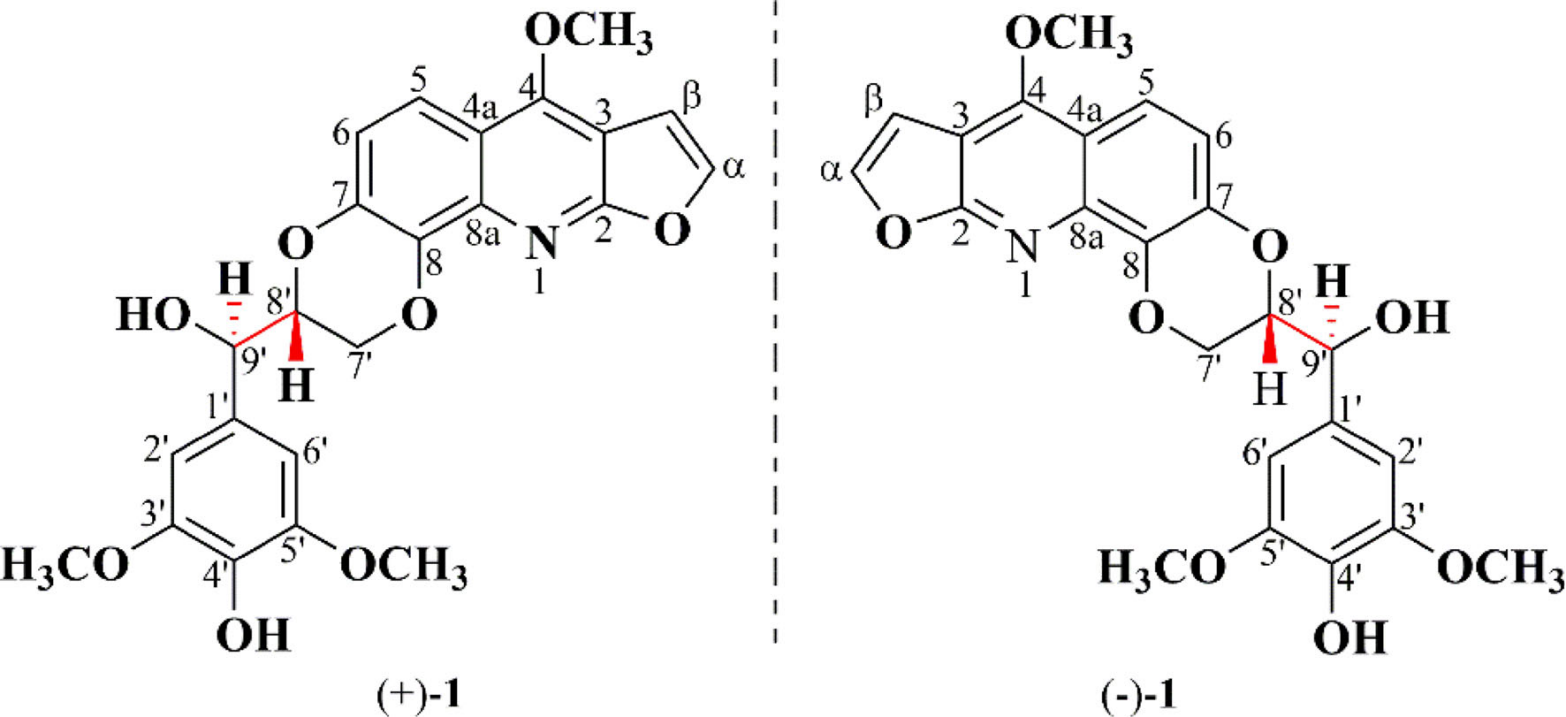
Chemical structures of (±)-zanthonitidine A (**1**)

The genus *Zanthoxylum* Linn of the Rutaceae family comprises of about 250 species in the world, mainly distributed in Asian, America, Africa, tropical and subtropical regions in Oceania. There are 39 species and 14 varietas in China [1], and the largest part occurs in south of the Yangtze River and south western provinces [2]. *Zanthoxylum nitidum* (Roxb.) DC. (Rutaceae) is a morphologically variable species with hooked prickly branchlets plant of the *Zanthoxylum* genus [3]. The radix of *Z. nitidum* was recorded as a traditional Chinese medicine, named “liangmianzhen” in Chinese Pharmacopeia (Version 2015), and has been widely used for the treatment of toothache, neuralgia, stomachache, sore throat, rheumatoid arthritis, turgescence and venomous snake bite [4]. It was main raw material of Chinese herbal toothpaste called “liangmianzhen”, and it is also used for some preparations, such as Jinji Tablet, Dieda Wanhua Oil. Several types of alkaloids including quinolines, isoquinolines, quinolones and benzophenanthridines, have previously been isolated from *Z. nitidum* [3, 5, 6], and some other kinds of compounds including coumarins and lignans, were also reported in this plant [5, 7]. Among them, alkaloids, especially benzophenanthridines, are considered as the main bioactive constituents, which show various pharmacological activity, including inhibiting DNA topoisomerase Ι [8], anti-inflammatory [6], anti-nociceptive [9], inhibiting the growth and inducing the pro-apoptosis [10]. With the purpose to discover more pharmacological alkaloids, we performed the phytochemical investigation on the radix of *Z. nitidum.* As a result, a pair of new enantiomeric furoquinoline alkaloids, (±)-zanthonitidine A (**1**) (Fig. 1), together with nine known alkaloids (**2**–**10**) (Fig. S1) were obtained. Herein, we report their isolation, structural elucidation, and antibacterial activity.

## Results and Discussion

Zanthonitidine A (**1**) was obtained as a yellow powder. Its molecular formula was determined by HRESIMS ([M + H]^+^, 440.13478, calcd. 440.13399) as C_23_H_21_NO_8_, implying fourteen degrees of unsaturation. The IR spectrum showed the absorptions at 3425 and 1624 cm^−1^, indicating the existence of hydroxyl and phenyl groups. The ^1^H NMR spectrum (Table 1) showed two pairs of AB doublets at *δ*_H_ 7.79 (1H, d, *J* = 9.2 Hz), 7.56 (1H, d, *J* = 2.3 Hz), 7.14 (1H, d, *J* = 9.2 Hz), 7.06 (1H, d, *J* = 2.3 Hz); two aromatic protons at *δ*_H_ 6.68 (2H, s); one methylene group at *δ*_H_ 3.86 (1H, dd, *J* = 12.6, 1.5 Hz), 3.71 (1H, dd, *J* = 12.6, 5.3 Hz); two methyne groups at 4.95 (1H, d, *J* = 8.1 Hz), 4.22 (1H, m); three methoxyl groups at *δ*_H_ 4.44 (3H, s), 3.91 (6H, s). The ^13^C NMR spectrum displayed 15 aromatic carbons at *δ*_C_ 163.8 (s), 158.2 (s), 147.7 (s), 147.7 (s), 143.8 (s), 137.0 (s), 135.8 (s), 135.8 (s), 127.2 (s), 116.5 (d), 115.1 (d), 114.6 (s), 104.6 (d), 104.6 (d), 102.8 (s); two olefinic carbons at *δ*_C_ 143.3 (d), 105.2 (d); one methylene group at *δ*_C_ 61.7 (t); two methyne groups at *δ*_C_ 79.4 (d), 77.4 (d); three methoxyl groups at *δ*_C_ 59.5 (q), 56.7 (q), 56.7 (q). Based on these data, **1** was presumed to be a furoquinoline containing glycerol and benzene moieties.

**Table 1:** ^1^H (600 MHz, *δ* in ppm, *J* in Hz) and ^13^C NMR (150 MHz, *δ* in ppm) data of zanthonitidine A (**1**) in CDCl_3_

Position	*δ*_H_ (m, *J*, Hz)	*δ*_C_
2		163.8
3		102.8
4		158.2
4a		114.6
5	7.79 (d, 9.2)	115.1
6	7.14 (d, 9.2)	116.5
7		143.8
8, 4′		135.8
8a		137.0
1′		127.2
2′, 6′	6.68 (s)	104.6
3′, 5′		147.7
7′a	3.71 (dd, 12.6, 1.5) 3.86 (dd, 12.6, 5.3)	61.7
7′b		
8′	4.22 (m)	79.4
9′	4.95 (d, 8.1)	77.4
α	7.56 (d, 2.3)	143.3
β	7.06 (d, 2.3)	105.2
4-OCH_3_	4.44 (s)	59.5
3′, 5′-OCH_3_	3.91 (s)	56.7

**Fig. 2:**
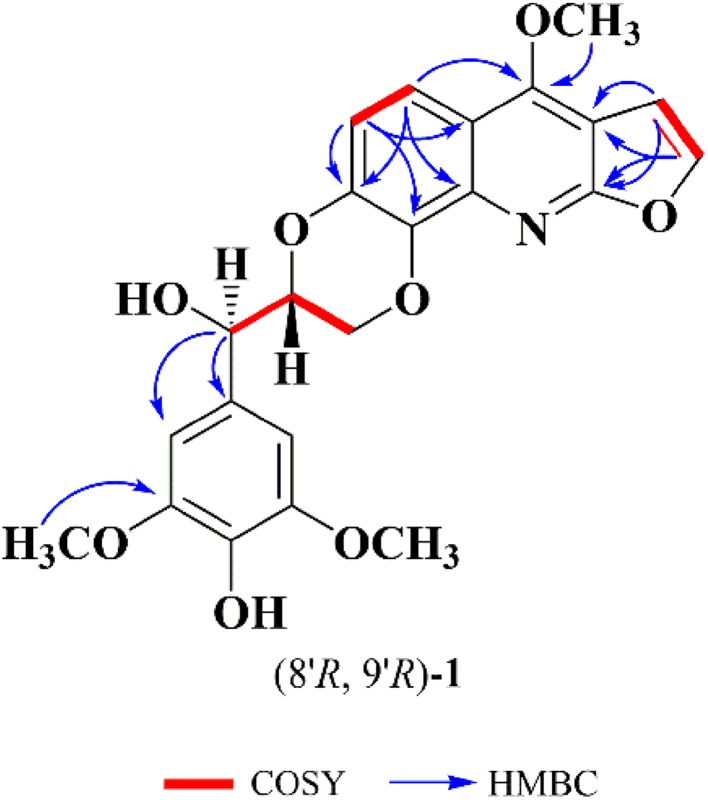
Key 2D NMR correlations of (±)-zanthonitidine A (**1**)

**Fig. 3:**
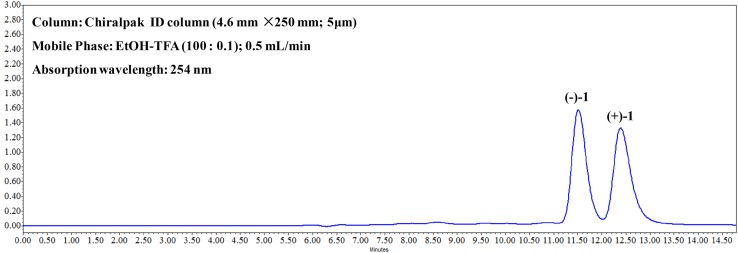
Chiral analysis of zanthonitidine A (**1**)

**Fig. 4:**
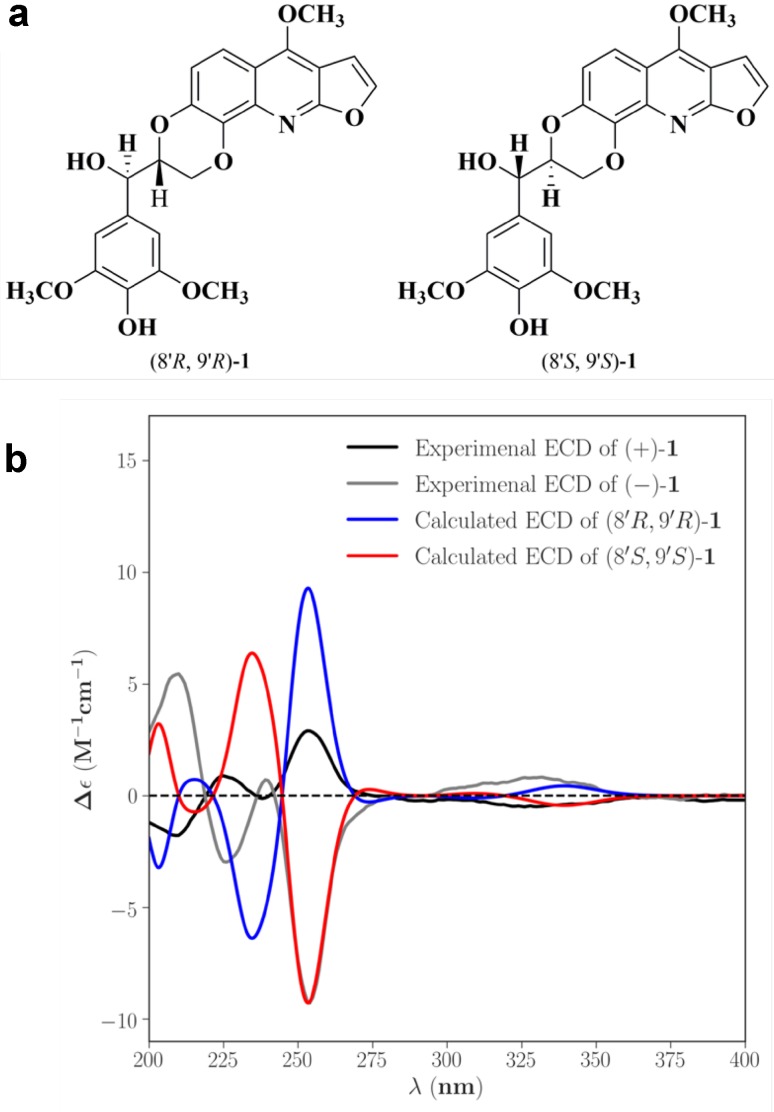
**a** Two possible stereochemical structures of **1**; **b** experimental ECD spectra of (+)-**1**/(−)-**1** and calculated ECD spectra of (8′*R*, 9′*R*)/(8′*S*, 9′*S*) of **1**

The structure was elucidated by detailed interpretation of 2D NMR correlations (Fig. 2). The HMBC correlations from *δ*_H_ 7.56 (H-*α*) to *δ*_C_ 163.8 (C-2) and *δ*_C_ 102.8 (C-3); from *δ*_H_ 7.06 (H-*β*) to *δ*_C_ 163.8 (C-2) and *δ*_C_ 102.8 (C-3); from *δ*_H_ 7.79 (H-5) to *δ*_C_ 158.2 (C-4), 143.8 (C-7), and 137.0 (C-8a); from *δ*_H_ 7.14 (H-6) to *δ*_C_ 143.8 (C-7), 135.8 (C-8), and 114.6 (C-4a); from *δ*_H_ 4.44 (4-OCH_3_) to *δ*_C_ 158.2 (C-4); together with the ^1^H-^1^H COSY correlations of H-*α*/H-*β* and H-5/H-6 gave a furoquinoline moiety. The HMBC correlations from *δ*_H_ 4.95 (H-9′) to *δ*_C_ 127.2 (C-1′) and *δ*_C_ 104.6 (C-2′); *δ*_H_ 3.91 (3′-OCH_3_) to *δ*_C_ 147.7 (C-3′); together with the ^1^H-^1^H COSY correlations of H-7′/H-8′ and H-8′/H-9′ suggested a glycerol segment at C-1′ position of benzene moiety. In consideration of the degrees of unsaturation and the chemical shift of *δ*_C_ 143.8 (C-7) and *δ*_C_ 135.8 (C-8), the C-O bonds should exist in C-7/C-8′ and C-8/C-7′, and formed a 1,4-dioxane moiety. Thus, the planar structure of **1** was established. There is no obvious absorption of electronic circular dichroism, and the coupling constant between the H-8′ and H-9′ was 8.1 Hz, which indicated that **1** was proposed to be a racemate mixture. Further Chiralpak ID column chromatography was performed, and obtained the enantiomers, (+)-**1** and (−)-**1** (Fig. 3). The absolute configurations of the enantiomers were then determined by comparing the experimental electronic circular dichroism (ECD) to the caculated ECD using the time-dependent density functional theory (TD-DFT) of the Gaussian 09 program package. The ECD spectra for (8′*R,* 9′*R*)-**1** and (8′*S,* 9′*S*)-**1** were calculated at the same theory level. The experimental ECD spectra of (+)-**1** and (−)-**1** resembled the calculated spectra of (8′*R,* 9′*R*)-**1** and (8′*S,* 9′*S*)-**1**, respectively (Fig. 4). Accordingly, the absolute configurations of (+)-**1** and (−)-**1** were then determined as (8′*R,* 9′*R*)-**1** and (8′*S,* 9′*S*)-**1**.

The known compounds were identified as 8-methoxy-*N*-methylflindersine (**2,** zanthobungeanine) [11], 4-methoxyfuro[2,3-b]quinoline-8-ol (**3,** robustine) [12], 4-methoxyfuro[2,3-b]-quinoline (**4,** dictamnine) [13], 4,8-dimethoxy-2-quinolone (**5,** edulitine) [14], 4,7,8-trimethoxyfuro[2,3-b]quinoline (**6**, skimmianine) [15], 4-methoxy-*N*-methyl-2-quinolone (**7**) [16], *trans*-(−)-9,10-dihydroxy-9,10-dihydrozanthobungeanine (**8,** zanthodioline) [17], 4,8-dimethoxyfuro[2,3-b]quinoline (**9,** *γ*-fagarine) [18] and 4-methoxyfuro[2,3-b]quinoline-8-*O*-*β*-d-glucopyranoside (**10**) [19] by comparing their spectroscopic data with those reported in the literatures.

The antimicrobial activities of compounds **1**–**9** was tested on the gram-positive strains *Bacillus subtilis*, *Enterococcus faecalis*, and *Staphylococcus aureus* (Table 2); penicillin was used as the positive control. Both compounds (+)-**1** and (−)-**1** showed moderate inhibitory activities against *Enterococcus* *faecalis* and *Staphylococcus aureus* with MIC values of 21.97, 21.97 μg/mL and 12.54, 25.09 μg/mL, respectively. Compounds **3**, **7** and **8** also showed inhibitory activities against *Enterococcus* *faecalis*.

**Table 2:** Antibacterial activity of compounds **1**–**9** (MIC, μg/mL)

Compounds	*Bacillus subtilis*	*Enterococcus faecalis*	*Staphylococcus aureus*
(+)-**1**	–^a^	21.97	21.97
(−)-**1**	–	12.54	25.09
**2**	–	–	–
**3**	–	5.37	–
**4**	–	–	–
**5**	–	–	–
**6**	–	–	–
**7**	–	18.91	–
**8**	–	37.83	–
**9**	–	–	–
Penicillin^b^	5.92	< 2.96	< 2.96

^a^Inactive (MIC > 50 μg/mL)

^b^Penicillin: positive control

In summary, the phytochemical investigation of the radix of *Zanthoxylum nitidum* in this study led to the identification of ten alkaloids (**1**–**10**) including a pair of new enantiomeric furoquinoline alkaloids, (±)-zanthonitidine A (**1**), and nine known ones. Biological assay for the antibacterial activities of **1**–**9** was performed, and the results showed that (+)-**1**, (−)-**1**, **3**, **7** and **8** possessed antibacterial activities.

## Experimental

### General Experimental Procedures

Optical rotations were measured with a Horiba SEPA-300 polarimeter. UV spectra were obtained using a Shimadzu UV-2401A spectrophotometer. CD spectra were tested using Chirascan Circular Dichroism spectrometer. A Tenor 27 spectrophotometer was used for scanning IR spectroscopy with KBr pellets. MS data were measured on Agilent G6230 TOF Mass spectrometer. 1D-NMR and 2D-NMR spectra were measured on a Bruker AM-400, DRX-500 or AVANCE III-600 at 298 K. Chemical shifts (*δ*) were expressed in parts per million (ppm) with reference to the solvent signals. Semi-preparative HPLC was performed on Waters HPLC system (1525 pump with 2998 photodiode array detector and 2707 autosampler) coupled with Zorbax Eclipse-C18 (9.4 mm × 250 mm; 5 μm) for purification or DAICEL Chiralpak ID column (4.6 mm × 250 mm; 5 μm) for chiral analysis. Column chromatography was performed on silica gel (100–200 mesh and 200–300 mesh, Qingdao Yu-Ming-Yuan Chemical Co. Ltd., Qingdao, China), Sephadex LH-20 (Pharmacia Fine Chemical Co., Uppsala, Sweden) or Lichroprep RP-18 gel (40–63 μm, Merck, Darmstadt, Germany). Thin layer chromatography (TLC) was performed on silica gel GF254 on glass plates (Qingdao Yu-Ming-Yuan Chemical Co. Ltd.) with detection by visualization with a UV lamp at 254 and 365 nm, and spots were visualized under ultra-violet light and 5% sulfuric acid–ethanol reagent.

### Plant Material

The radix of *Zanthoxylum nitidum* (Roxb.) DC. (Rutaceae) was purchased from Bozhou Herbal Medicine Market (Anhui, China) in December 2016 and authenticated by Prof. Min-Jian Qin of China Pharmaceutical University. A voucher specimen was deposited in the Herbarium of China Pharmaceutical University.

### Extraction and Isolation

The air-dried and milled radix of *Z. nitidum* (10 kg) was extracted three times with methanol (3 × 20 L) under reflux, and the resulting solution was evaporated under reduced pressure to yield the methanol extract (688.4 g). All amount of the extract was separated by a silica gel column chromatography (CC) (100–200 mesh), eluted with a gradient of CHCl_3_–MeOH (100:0, 95:5, 9:1, 8:2, 7:3, 1:1, 0:1) to yield eleven fractions (Fr. 1–Fr. 11). Fr. 3 (23.7 g) was subjected to silica gel CC (200–300 mesh), eluted with a gradient of petroleum ether-acetone (100:0, 30:1, 10:1, 7:1, 3:1, 1:1) to yield eight subfractions (Fr. 3–1 to Fr. 3–8). Fr. 3.5 (10.0 g) was further purified by RP-18 gel CC (20–100% MeOH–H_2_O), Sephadex LH-20 CC (CHCl_3_–MeOH, 1:1), and silica gel CC (200–300 mesh) (petroleum ether-chloroform, 1:3) to afford 8-methoxy-*N*-methylflindersine (**2**) (20.1 mg), 4-methoxyfuro[2,3-b]quinoline-8-ol (**3**) (18.0 mg), and 4,7,8-trimethoxyfuro[2,3-b]quinoline (**6**) (22.5 mg). Fr. 3.6 (5.1 g) was further purified by RP-18 gel CC (20–100% MeOH–H_2_O) and Zorbax Eclipse C18 column (41% acetonitrile–H_2_O) to afford 4-methoxy-*N*-methyl-2-quinolone (**7**) (10.8 mg), *trans*-(−)-9,10-dihydroxy-9,10-dihydrozanthobungeanine (**8**) (17.8 mg), and 4,8-dimethoxyfuro[2,3-b]quinoline (**9**) (17.0 mg). Fr. 3.7 (7.2 g) was further separated by RP-18 gel CC (20–100% MeOH–H_2_O) to yield five subfractions (Fr. 3–7–1 to Fr. 3–7–5). 4,8-dimethoxy-2-quinolone (**5**) (15.6 mg) was crystallized out of Fr. 3–7–2. Fr. 3–7–3 was subjected to Sephadex LH-20 CC (CHCl_3_–MeOH, 1:1) and then purified by Zorbax Eclipse C18 column (70% acetonitrile–H_2_O) to get zanthonitidine A (**1**) (2.7 mg) which was further separated by a chiralpak ID column (EtOH–TFA, 100:0.1) to yield (+)-**1** (0.1 mg) and (−)-**1** (0.2 mg). Fr. 6 (25.1 g) was subjected to Sephadex LH-20 CC (CHCl_3_–MeOH, 1:1), and then further purified by Zorbax Eclipse C18 column (60% acetonitrile–H_2_O) to give 4-methoxyfuro[2,3-b]-quinoline (**4**) (15.1 mg) and 4-methoxyfuro[2,3-b]quinoline-8-*O*-*β*-d-glucopyranoside (**10**) (8.3 mg).

#### Zanthonitidine A (**1**)

Yellow powder; UV (MeOH) *λ*_max_ (log ε) 207.5 (4.67), 254.5 (4.86), 323.0 (3.80), 407.5 (2.62), 589.5 (2.58) nm; IR (KBr) *ν*_max_ 3425, 2928, 1624, 1516, 1487, 1461, 1409, 1370, 1341, 1298, 1260, 1238, 1156, 1097, 1060, 979 cm^−1^; ^1^H (600 MHz) and ^13^C (150 MHz) NMR data, see Table 1; ESIMS (positive): *m*/*z* 440.33 [M + H]^+^; HRESIMS (positive): *m*/*z* 440.13478 (calcd for C_23_H_22_NO_8_, 440.13399).

#### (+)-Zanthonitidine A ((+)-**1**)

Yellow powder; [*α*]_D_^20^ +20.0 (*c* 0.02, MeOH); ECD (0.23 mM, MeOH) *λ*_max_ (Δε) 207 (− 2.5), 213 (− 2.2), 223 (1.2), 239 (− 0.8), 254 (4.0) nm; HRESIMS (positive): *m/z* 440.13399 (calcd for C_23_H_22_NO_8_, 440.13399).

#### (−)-Zanthonitidine A ((−)-**1**)

Yellow powder; [*α*]_D_^20^ -93.3 (*c* 0.01, MeOH); ECD (0.46 mM, MeOH) *λ*_max_ (Δε) 203 (4.3), 210 (7.1), 226 (− 3.6), 241 (2.0), 253 (− 11.0) nm; HRESIMS (positive): *m/z* 440.13338 (calcd for C_23_H_22_NO_8_, 440.13399).

### ECD Calculation

Conformational analysis was initially performed using Confab at MMFF94 force field for two configurations for **1**. Room-temperature equilibrium populations were calculated according to Boltzmann distribution law. The conformers with Boltzmann-population of over 1% were subjected to ECD calculations. The theoretical calculation was carried out using Gaussian 09 [20]. The comformers was initially optimized at PM6 using semiempirical theory method, and then optimized at the B3LYP/6-311G (d, p) in MeOH using the IEFPCM model. The theoretical calculation of ECD was conducted in MeOH using Time-dependent Density Functional Theory (TD-DFT) at the same theory level.

### Antibacterial Assay

*Bacillus subtilis, Enterococcus faecalis,* and *Staphylococcus aureus* were cultured in Luria–Bertani (LB) broth. The broth microdilution assay was applied for the antibacterial activity screening according to CLSI guidelines (CLSI 2015). *Bacillus subtilis*, *E. faecalis*, and *S. aureus* were propagating in the Mueller–Hinton broth (0.20%, *w*/*v*, beef extract; 1.75%, *w*/*v,* acid digest of casein; 0.15%, *w*/*v*, starch). After incubation with various concentrations of **1**–**9** at 37 °C for 24 h, the 96-well plates were checked by visual inspection; penicillin was used as the positive control. The MICs were determined as the lowest concentration for no visible growth of bacteria.

## Electronic supplementary material

Below is the link to the electronic supplementary material.

Supplementary material 1 (DOCX 1301 kb)

## References

[1] Yuan HM, Qiu L, Xie ZJ, Zou L, Zheng J, Fu Q (2015). Chin. J. Chin. Mat. Med..

[2] Liu YY, Cao W, Zhang Y, Wang SW (2012). Chin. J. Ethnomed. Ethnopharm..

[3] Kong DY, Gray AI, Hartley TG, Waterman PG (1996). Biochem. Syst. Ecol..

[4] Editorial Board of ‘Zhonghua Bencaoʼ, State Administration of Traditional Chinese Medicine of the Peopleʼs Republic of China, Zhonghua Bencao; Shanghai Scientific and Technical Publishing House: Shanghai, **4**, 3821 (1999)

[5] Yang CH, Cheng MJ, Lee SJ, Yang CW, Chang HS, Chen IS (2009). Chem. Biodiv..

[6] Hu J, Zhang WD, Liu RH, Zhang C, Shen YH, Li HL, Liang MJ, Xu XK (2006). Chem. Biodiv..

[7] Shen JW, Zhang XF, Peng SL, Ding LS (2005). Nat. Prod. Res. Dev..

[8] Fang SD, Wang LK, Hecht SM (1993). J. Org. Chem..

[9] Hu J, Shi XD, Mao X, Chen JG, Zhu L, Zhao QJ (2013). J. Ethnopharmacol..

[10] Fang ZQ, Tang YQ, Jiao W, Xing ZQ, Guo ZX, Wang WC, Xu ZH, Liu ZX (2014). Food Chem. Toxicol..

[11] Joshi BS, Moore KM, Pelletier SW (1991). Phytochem. Anal..

[12] Chlouchi A, Girard C, Tillequin F, Bévalot F, Waterman PG, Muyard F (2006). Biochem. Syst. Ecol..

[13] Liu QW, Tan CH, Qu SJ, Fan X, Zhu DY (2006). Chin. J. Nat. Med..

[14] Imai F, Iton K, Kishibuchi N, Kinoshita T, Sankawa U (1989). Chem. Pharm. Bull..

[15] Ratheesh M, Sindhu G, Helen A (2013). Inflamm. Res..

[16] Min YD, Kwon HC, Yang MC, Lee KH, Choi SU, Lee KR (2007). Arch. Pharm. Res..

[17] Chen IS, Tsai IW, Teng CM, Chen JJ, Chang YL, Ko FN, Lu MC (1997). Phytochemistry.

[18] Tang J, Zhu W, Tu ZB (1995). Chin. Trad. Herb. Drugs.

[19] Liu J, Li CJ, Ni L, Yang JZ, Li L, Zang CX, Bao XQ, Zhang D, Zhang DM (2015). RSC Adv..

[20] Frisch MJ, Trucks GW, Schlegel HB, Scuseria GE, Robb MA, Cheeseman JR, Scalmani G, Barone V, Mennucci B, Petersson GA, Nakatsuji H, Caricato M, Li X, Hratchian HP, Izmaylov AF, Bloino J, Zheng G, Sonnenberg JL, Hada M, Ehara M, Toyota K, Fukuda R, Hasegawa J, Ishida M, Nakajima T, Honda Y, Kitao O, Nakai H, Vreven T, Montgomery JA, Peralta JE, Ogliaro F, Bearpark M, Heyd JJ, Brothers E, Kudin KN, Staroverov VN, Keith T, Kobayashi R, Normand J, Raghavachari K, Rendell A, Burant JC, Iyengar SS, Tomasi J, Cossi M, Rega N, Millam JM, Klene M, Knox JE, Cross JB, Bakken V, Adamo C, Jaramillo J, Gomperts R, Stratmann RE, Yazyev O, Austin AJ, Cammi R, Pomelli C, Ochterski JW, Martin RL, Morokuma K, Zakrzewski VG, Voth GA, Salvador P, Dannenberg JJ, Dapprich S, Daniels AD, Farkas O, Foresman JB, Ortiz JV, Cioslowski J, Fox DJ (2010). GAUSSIAN 09 (Revision B.01).

