# Supplementary material for: (±)-Zanthonitidine A, a Pair of Enantiomeric Furoquinoline Alkaloids from Zanthoxylum nitidum with Antibacterial Activity
Source: Nat Prod Bioprospect. 2018 May 31;8(5):361–7. doi: 10.1007/s13659-018-0169-7 (PMC6109446; doi:10.1007/s13659-018-0169-7)
Supplement: Supplementary file 1 — Supplementary material 1 (DOCX 1301 kb) [file 13659_2018_169_MOESM1_ESM.docx]

**Supplementary Information**

**(±)-Zanthonitidine A, a pair of enantiomeric furoquinoline alkaloids from** ***Zanthoxylum nitidum* with antibacterial activity**

Li-Na Zhao^a,1^, Xi-Xi Guo^a,1^, Shuai Liu^b^, Li Feng^a^, Qi-Rui Bi^a^, Zhe Wang^a,^^*^, Ning-Hua Tan^a,*^

^a^ Department of TCMs Pharmaceuticals, School of Traditional Chinese Pharmacy, China Pharmaceutical University, Nanjing 211198, China

^b^ Faculty of Life Science and Food Engineering, Huaiyin Institute of Technology, Huaian 223001, China

^1^ These authors contributed equally to this work.

* **Corresponding Authors**

Tel: + 86-25-84155947. E-mail addresses: wangzhe@cpu.edu.cn (Z. Wang), nhtan@cpu.edu.cn (N.-H. Tan).

**Table of Contents**

**Fig. S1** Chemical structures of **2**-**10**

**Fig. S2** ^1^H NMR spectrum of zanthonitidine A (**1**)

**Fig. S3** ^13^C NMR spectrum of zanthonitidine A (**1**)

**Fig. S4** HSQC spectrum of zanthonitidine A (**1**)

**Fig. S5** ^1^H-^1^H COSY spectrum of zanthonitidine A (**1**)

**Fig. S6** HMBC spectrum of zanthonitidine A (**1**)

**Fig. S7** ESIMS spectrum of zanthonitidine A (**1**)

**Fig. S8** HRESIMS spectrum of zanthonitidine A (**1**)

**Fig. S9** HRESIMS spectrum of (+)-zanthonitidine A ((+)-**1**)

**Fig. S10** HRESIMS spectrum of (-)-zanthonitidine A ((-)-**1**)

**Fig. S11** UV spectrum of zanthonitidine A (**1**)

**Fig. S12** IR spectrum of zanthonitidine A (**1**)

**Table S1** Energies of the dominative conformers of zanthonitidine A (**1**) at MMFF94 force field

**Table S2** Energies of the conformers of zanthonitidine A (**1**) at B3LYP/6-311G (d, p) in methanol

**Fig. S1** Chemical structures of **2**-**10**

**

**

**Fig. S2** ^1^H NMR spectrum of zanthonitidine A (**1**)

**Fig. S3** ^13^C NMR spectrum of zanthonitidine A (**1**)

**Fig. S4** HSQC spectrum of zanthonitidine A (**1**)

**Fig. S5** ^1^H-^1^H COSY spectrum of zanthonitidine A (**1**)

**Fig. S6** HMBC spectrum of zanthonitidine A (**1**)

**Fig. S7** ESIMS spectrum of zanthonitidine A (**1**)

**Fig. S8** HRESIMS spectrum of zanthonitidine A (**1**)

**Fig. S9** HRESIMS spectrum of (+)-zanthonitidine A ((+)-**1**)

**Fig. S10** HRESIMS spectrum of (-)-zanthonitidine A ((-)-**1**)

**Fig. S11** UV spectrum of zanthonitidine A (**1**)

**Fig. S12** IR spectrum of zanthonitidine A (**1**)

**Table S1** Energies of dominative conformers of zanthonitidine A (**1**) at MMFF94 force field

| Compound | Conformer | Energy (kcal/mol) | Population (%) |
| --- | --- | --- | --- |
| (8’*S*, 9’*S*)-1 | 1 | 145.04 | 92.78 |
| (8’*S*, 9’*S*)-1 | 2 | 146.84 | 4.45 |
| (8’*S*, 9’*S*)-1 | 3 | 147.62 | 1.20 |
| (8’*S*, 9’*S*)-1 | 4 | 147.67 | 1.09 |
| (8’*R*, 9’*R*)-1 | 1 | 147.30 | 77.18 |
| (8’*R*, 9’*R*)-1 | 2 | 148.54 | 9.51 |
| (8’*R*, 9’*R*)-1 | 3 | 148.68 | 7.43 |
| (8’*R*, 9’*R*)-1 | 4 | 149.00 | 4.38 |
| (8’*R*, 9’*S*)-1 | 1 | 147.79 | 69.62 |
| (8’*R*, 9’*S*)-1 | 2 | 148.48 | 21.45 |
| (8’*R*, 9’*S*)-1 | 3 | 149.16 | 6.86 |
| (8’*S*, 9’*R*)-1 | 1 | 147.79 | 89.03 |
| (8’*S*, 9’*R*)-1 | 2 | 149.36 | 6.31 |
| (8’*S*, 9’*R*)-1 | 3 | 149.86 | 2.71 |

**Table S2** Energies of dominative conformers of zanthonitidine A (**1**) at B3LYP/6-311G (d, p) in methanol

| Configuration | Conformer | Structure | E (Hartree) | E (kcal/mol) | Population (%) |
| --- | --- | --- | --- | --- | --- |
| (8’*S*, 9’*S*)-1 | 4 | 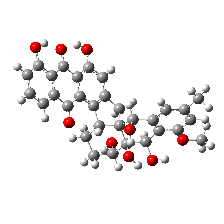 | -1545.98020005 | -970117.21 | 100 |
| (8’*R*, 9’*R*)-1 | 4 | 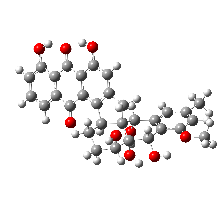 | -1545.98020005 | -970117.21 | 100 |
| (8’*R*, 9’*S*)-1 | 1 | 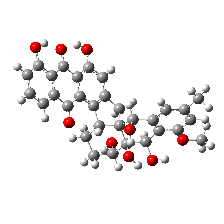 | -1545.97967365 | -970116.88 | 55.05 |
| (8’*R*, 9’*S*)-1 | 2 | 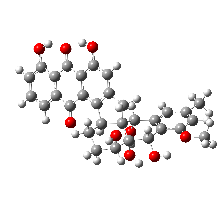 | -1545.97948224 | -970116.76 | 44.95 |
| (8’*S*, 9’*R*)-1 | 1 | 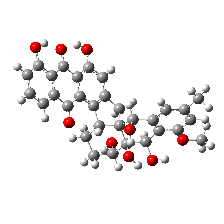 | -1545.97967365 | -970116.88 | 55.05 |
| (8’*S*, 9’*R*)-1 | 2 | 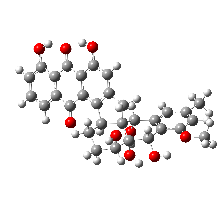 | -1545.97948224 | -970116.76 | 44.95 |
